# The diagnostic value of microrna-499 in acute myocardial infarction: A systematic review and meta-analysis

**DOI:** 10.5937/jomb0-52617

**Published:** 2025-03-21

**Authors:** Hong Du, Zhiyuan Zhang, Feifei Yan, Zichao Dong

**Affiliations:** 1 No.988 Hospital of Joint Logistics Support Force, Department of Cardio Thoracic Surgery, Jiaozuo, China; 2 Wuhan Asian Heart Hospital, Department of Cardio Surgery, Wuhan, China

**Keywords:** microRNA-499, acute mocardial infarction, diagnostic value, meta-analysis, mikroRNA-499, akutni infarkt mikarda, dijagnostička vrednost, meta-analiza

## Abstract

**Background:**

The objective of this study was to assess the diagnostic efficacy of MicroRNA-499 in cases of acute myocardial infarction.

**Methods:**

On May 6, 2023, four electronic databases PubMed, Embase, Web of Science, and Cochrane Library were searched with no time restriction. Quality assessment was conducted using Quality Assessment of Diagnostic Accuracy Studies-2 (QUADAS-2). Random-effects metaanalysis was employed to combine sensitivity, specificity, positive likelihood ratio (PLR), negative likelihood ratio (NLR), and diagnostic odds ratio (DOR). The evaluation of publication bias was conducted through the utilization of Deeks' asymmetry test on the funnel plot.

**Results:**

The studies that were included in the analysis, encompassing sample sizes ranging from 63 to 1155, exhibited a pooled sensitivity of 0.88 and a specificity of 0.97. The pooled PLR was determined to be 29.78, while the NLRwas found to be 0.13. Furthermore, the pooled DOR was calculated to be 236.10. The area under the curve (AUC) was reported as 0.98.

**Conclusions:**

MicroRNA-499 exhibits high sensitivity and specificity for diagnosing Acute Myocardial Infarction, indicating its potential as a valuable diagnostic biomarker.

## Abstract (sr)

**Uvod:**

Cilj ove studije je bio da se proceni dijagnostička efikasnost MicroRNA-499 u slučajevima akutnog infarkta miokarda.

**Metode:**

6. maja 2023. pretražene su četiri elektronske baze podataka PubMed, Embase, Web of Science i Cochrane Library bez vremenskog ograničenja. Procena kvaliteta je sprovedena korišćenjem Studije o proceni tačnosti dijagnostike-2 (KUADAS-2). Meta-analiza slučajnih efekata je korišćena da se kombinuju osetljivost, specifičnost, odnos pozitivne verovatnoće (PLR), odnos negativne verovatnoće (NLR) i dijagnostički odnos verovatnoće (DOR). Procena pristrasnosti publikacije je sprovedena korišćenjem Deeksovog testa asimetrije na dijagramu levka.

**Rezultati:**

Studije uključene u analizu, koje su obuhvatale uzorke od 63 do 1155, pokazale su zbirnu osetljivost od 0,88 i specifičnost od 0,97. Utvrđeno je da je objedinjeni PLR 29,78, dok je utvrđeno da je NLR 0,13. Štaviše, izračunato je da je objedinjeni DOR 236,10. Površina ispod krive (AUC) je prijavljena kao 0,98.

**Zaključak:**

MicroRNA-499 pokazuje visoku osetljivost i specifičnost za dijagnostikovanje akutnog infarkta miokarda, što ukazuje na njen potencijal kao vredan dijagnostički bio-marker.

## Introduction

Acute myocardial infarction (AMI) is a leading cause of mortality worldwide. Prompt and precise diagnosis of AMI is crucial for therapeutic interventions and prognostic determinations, effectively mitigating the incidence and mortality rate of AMI [1]. In clinical practice, circulating biomarkers of myocardial injury, such as cardiac troponin T, are the most effective and commonly used tools for the diagnosis of AMI, maximizing the benefits of revascularization therapy [2]. However, their diagnostic accuracy remains relatively low within the first 4 to 8 hours following the onset of AMI. Additionally, increased levels of cardiac troponin T (cTnT) and creatine kinase MB can be observed regardless of the occurrence of AMI, presenting challenges in distinguishing AMI from other conditions that may elevate these markers. Moreover, prior studies have demonstrated that significant elevations in cTnT are only detectable around 6 hours post-AMI, indicating the necessity for the discovery of novel and more reliable biomarkers for AMI diagnosis [3].

MicroRNAs (miRNAs) are a type of non-coding RNA that are small in size (19–25 nucleotides). They play a crucial role in regulating various biological processes. Literature has shown that the distribution of miRNAs in cells exhibits tissue and cell-specific patterns. Recent studies have revealed the presence of miRNAs in serum or plasma, suggesting their potential role as biological markers for cardiovascular diseases [4] [5]. MicroRNA-499, a member of the miRNA family, is expressed in the myocardium and skeletal muscle of mammals [6]. Preliminary studies have demonstrated an elevation in plasma or serum levels of miRNA-499 in patients with AMI. This association implies that miRNA-499 could potentially serve as a biomarker for AMI and a therapeutic indicator in clinical practice [7] [8].

Given the small sample sizes and the controversies surrounding most existing studies on miRNA-499 as a biomarker for AMI. Our goal is to provide a more comprehensive understanding of the role of miRNA- 499 in AMI diagnosis, thus contributing to the optimization of diagnostic strategies for AMI. We aspire to bridge the gap in current research and open a new horizon for the early detection and treatment of AMI.

## Materials and methods

The methodological approach employed in this article adhered to the guidelines outlined in the Preferred Reporting Items for Systematic Reviews and Meta-Analyses diagnostic test accuracy (PRISMA-DTA) guideline [9]. Since the information utilized in this article was obtained from published sources, neither informed consent nor ethical approval were required. Two researchers conducted a systematic search for relevant studies, independently determined their eligibility, collected information, and assessed the research’s quality. The two researchers were required to come to an agreement and resolve any points of contention.

### Search strategy

On May 6, 2023, four electronic databases PubMed, Embase, Web of Science, and Cochrane Library were searched with no time restriction. The grammar and vocabulary were tailored specifically to the database. The specific search terms were: (miRNA-499 OR microRNA-499) AND (AMI OR Acute Myocardial Infarction OR Heart Attack OR Cardiac Infarction OR Myocardial Infarct) AND (Diagnosis OR Diagnostic OR Diagnostic Use OR Diagnostic Value OR Biomarkers, Diagnostic OR Diagnostic Techniques, Cardiovascular OR Cardiac Biomarkers) AND (Sensitivity and Specificity OR ROC Curve OR Area Under Curve). No language restriction was imposed.

### Inclusion criteria

Studies included in the systematic review needed to meet the following criteria: 1) Study type: Published diagnostic trials concerning the accuracy of miRNA-499 in diagnosing AMI. 2) The subjects of the experimental group should be clinically diagnosed AMI patients while the subjects of the control group should be healthy people or patients with non-AMI coronary heart disease or patients with other cardiovascular diseases. 3) The miRNA-499 in blood and serum should be detected via Reverse Transcription Polymerase Chain Reaction. 4) The reported data must enable the calculation of the four-fold table related indicators. Exclusion Criteria: 1) Reviews, commentaries, conference proceedings, and case reports. 2) Studies involving animal experiments. 3) Studies with fewer than 10 cases. 4) Studies with incomplete data, errors, or from which data cannot be extracted.

### Data extraction

Data extraction will be independently conducted by two researchers, each screening the included literature and extracting relevant data. Upon completion of data extraction, the researchers will cross-verify the extracted content, which includes but is not limited to the authors’ names, countries, publication dates, sample sizes and correlation outcome data. In the event of any discrepancies between the two researchers’ findings, a third researcher will review the points of disagreement and make the final decision on whether the data should be included in the study. This process ensures a high level of reliability and accuracy in the data extracted.

### Quality assessment

Two researchers will independently assess the quality of the literature included in the study by utilizing the Quality Assessment of Diagnostic Accuracy Studies (QUADAS) tool [10]. The tool comprises 14 items, each of which is scored as »yes,« »no,« or »unclear.« These items cover various aspects of quality assessment, Any discrepancies in the quality assessment will be resolved through discussion or the involvement of a third researcher, if necessary. This ensures that only high-quality, reliable studies are included.

### Statistical analyses

Statistical analyses will be conducted using Stata 17.0 software. We pool the diagnostic data of each article and then combine them using random effects models. Heterogeneity among studies will be assessed using the Chi-square test, with the Q test and I^2^ statistic used for evaluating the degree of heterogeneity. Using chi-square statistics and I2 values, the heterogeneity between experiments was evaluated. Using the I2 statistic, heterogeneity was assessed among the included studies. A value of 0% for I2 indicated that there was no observed heterogeneity, whereas values > 50% showed significant variability. Publication bias will be evaluated using Deeks’ funnel plot, a graphical tool that can help to detect any potential bias or systematic heterogeneity among the results of different studies included in the meta-analysis.

## Results

### Search results and study selection

**Figure 1:**
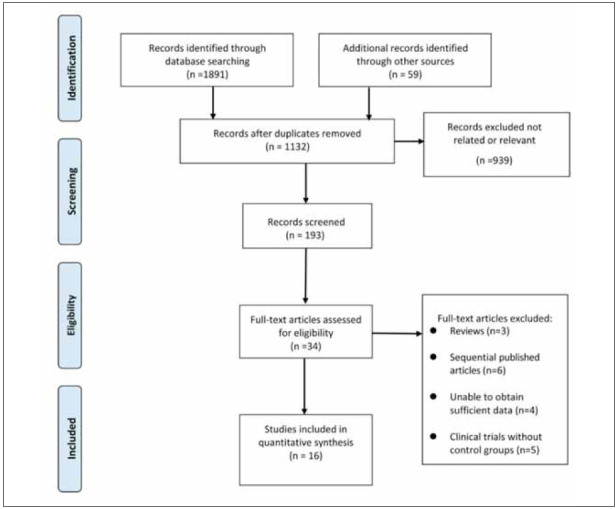
Selection process of included studies.

From the initial search of the electronic databases, 1950 related literatures were initially found. After removing repetitive literatures, 34 related literatures were obtained by reading titles and abstracts, and then 18 were excluded from further reading. Finally, 16 articles were included as shown in Figure 1 [11] [12] [13] [14] [15] [16] [17] [18] [19] [20] [21] [22] [23] [24] [25] [26].

### Study characteristics

The meta-analysis incorporated 16 diagnostic trials conducted from 2010 to 2018. These studies were sourced from various international locales and were executed by a diverse group of researchers. The sample sizes across the 16 studies varied considerably, ranging from 63 to 1155. The detailed characteristics of each study, including these metrics, are presented in Table 1.

**Table 1:** Characteristics of studies included in the meta-analysis. Abbreviations: cTnT, cardiac troponin T; CKMB, creatine kinase-MB.

Author	Year	Country	Sample type	Detection index	Experimental <br>group/n	Control <br>group/n
Fawzy [22]	2018	Greece	plasma	miR-499/cTnT	80	50
Liu [23]	2018	China	plasma	miR-499	145	30
Agiannitopoulos [21]	2017	Egypt	serum	miR-499	110	121
Shalaby [20]	2016	Egypt	serum	miR-499	48	25
Devaux [15]	2015	Luxembourg	plasma	miR-499/cTnT	224	931
Ji [16]	2015	China	serum	miR-499/cTnT	98	23
Liu [17]	2015	China	plasma	miR-499/cTnT	70	72
Zhao [18]	2015	China	plasma	miR-499/cTnT	59	60
Zhang [19]	2015	China	plasma	miR-499/cTnT	142	85
Olivieri [26]	2013	Italy	serum	miR-499/cTnT	31	32
Li CJ [25]	2013	China	serum	miR-499/CKMB	117	100
Gidlof [24]	2013	Sweden	plasma	miR-499/cTnT	319	88
Li YQ [14]	2013	China	plasma	miR-499/cTnT	67	32
Devaux [13]	2012	Luxembourg	plasma	miR-499	510	84
Corsten [11]	2010	Luxembourg	plasma	miR-499	32	36
Wang [12]	2010	China	plasma	miR-499	33	33

### Results of quality assessment

**Figure 2:**
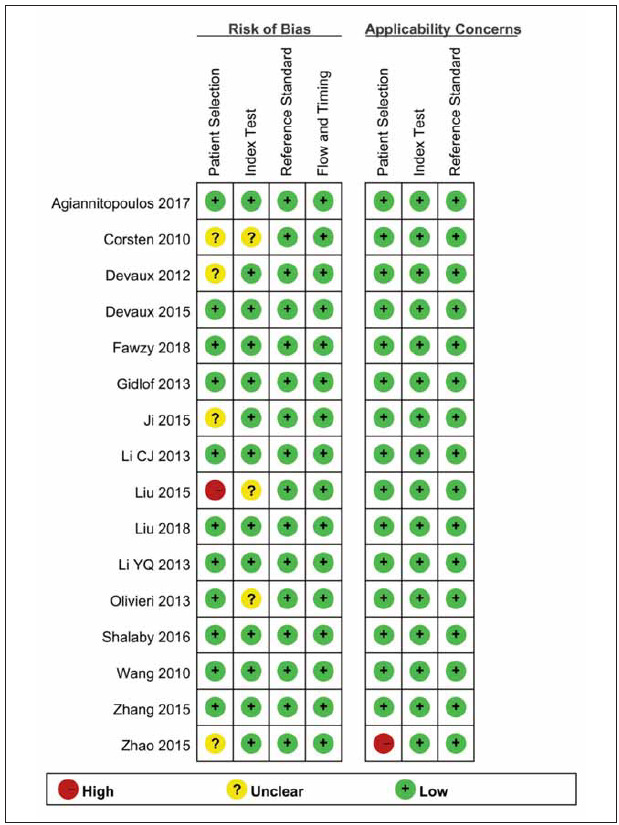
Quality assessment of included studies using Quality Assessment of Diagnostic Accuracy Studies-2 (QUADAS-2) tool criteria.

Figure 2 displays the QUADAS-2 evaluations for each of the studies included in this meta-analysis. The findings suggest that the quality of the studies included in each key domain was satisfactory, as the proportion of high risk was found to be less than 5%.

### Diagnostic value of miRNA-499 for acute myocardial infarction

**Figure 3:**
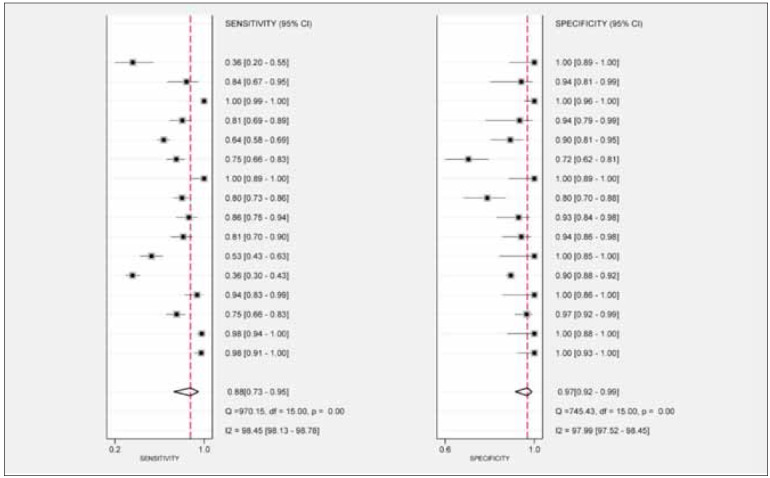
Forest plots of the sensitivity and specificity of miRNA-499 for acute myocardial infarction.

**Figure 4:**
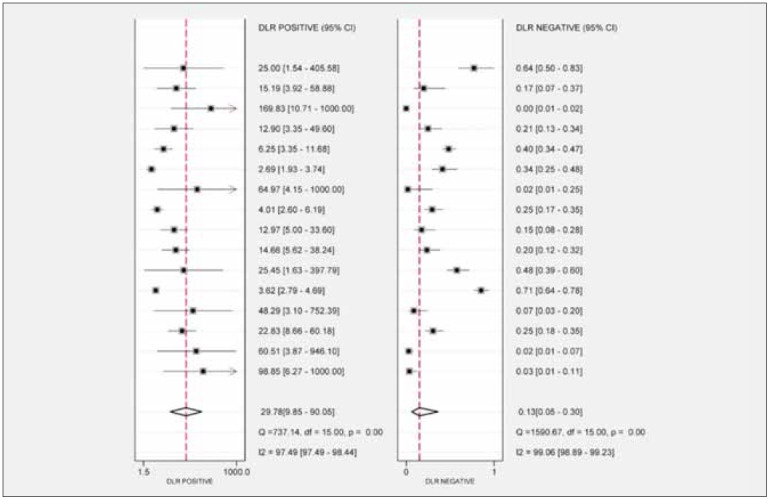
Forest plots of the PLR and NLR of miRNA-499 for acute myocardial infarction.

**Figure 5:**
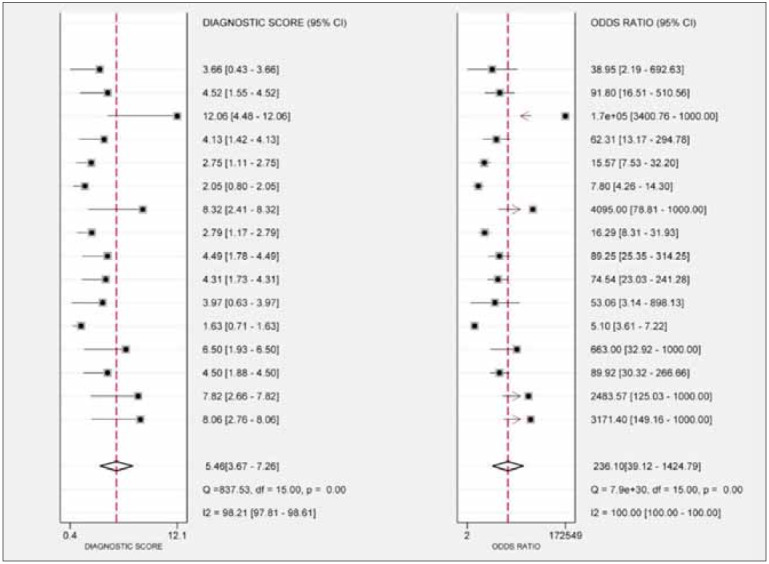
Forest plots of the diagnostic score and diagnostic odds ratio of miRNA-499 for acute myocardial infarction.

**Figure 6:**
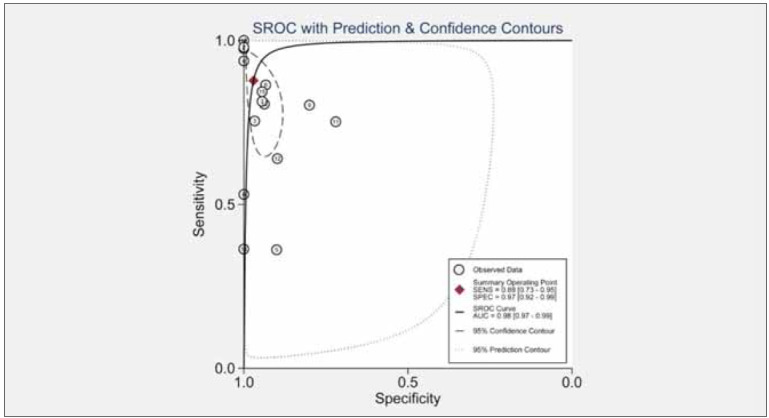
The ROC of miRNA-499 for acute myocardial infarction.

The meta-analysis of the 16 included studies demonstrated substantial heterogeneity for both sensitivity (I^2^=98.13%, 95% CI [98.13, 98.78]) and specificity (I^2^=97.99%, 95% CI [97.52, 98.45]), both exceeding 50%. Therefore, a random-effects model was utilized. The pooled sensitivity (SEN) was 0.88 (95% CI [0.73, 0.95]), and the pooled specificity (SPE) was 0.97 (95% CI [0.92, 0.99]) (Figure 3). The pooled positive likelihood ratio (PLR) was 29.78 (95% CI [9.85, 90.05]), and the pooled negative likelihood ratio (NLR) was 0.13 (95% CI [0.05, 0.30]) (Figure 4). The pooled diagnostic odds ratio (DOR) was 236.10 (95% CI [39.12, 1424.79]) (Figure 5). The area under the curve (AUC) was 0.98 (95% CI [0.97, 0.99]) (Figure 6).

### Publication bias

**Figure 7:**
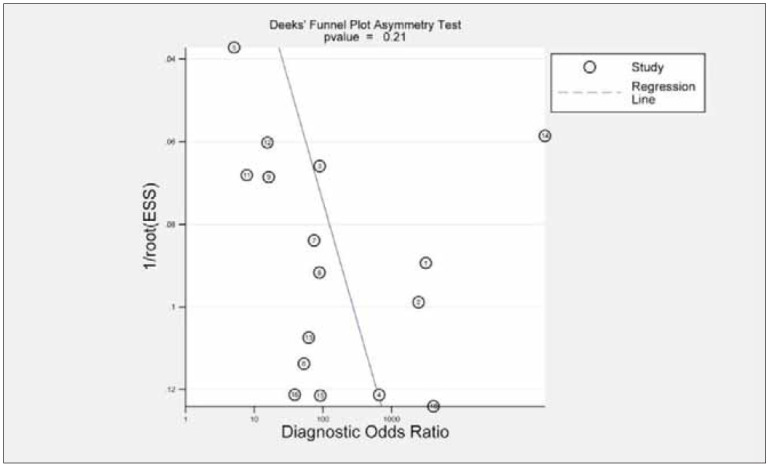
Funnel plot for publication bias in all included studies.

The funnel plots created using the gathered data exhibited a balanced distribution, indicating the absence of substantial publication bias (p=0.21, Figure 7).

## Discussion

Acute myocardial infarction occurs as a consequence of acute inadequate blood flow and oxygen supply to the coronary arteries, leading to necrosis of the myocardium. Clinically, AMI often presents with severe and enduring chest pain behind the sternum, which cannot be significantly alleviated by rest or nitrate drugs [1] [27]. Complications such as arrhythmias and shock, posing significant threats to life. Traditional clinical biomarkers, such as cTnT and CK-MB, are widely utilized to diagnose AMI. However, their diagnostic accuracy remains comparatively low within the initial 4–8 hours following symptom onset. Thus, the identification of novel biomarkers to enhance AMI diagnosis and improve therapeutic outcomes is imperative [28] [29].

One such promising biomarker is circulating miRNA-499, which has been shown to effectively distinguish between AMI and non-AMI cases. A previous study revealed a connection between the expression levels of circulating miRNA-499 and AMI [30], suggesting its potential as a clinical biomarker. However, this study focused exclusively on the Asian population and did not include a comprehensive collection of relevant literature. To overcome these limitations, our current systematic review and meta-analysis incorporated 16 diagnostic trials from various countries with a QUADAS score above 9, providing valuable insights into the role of miRNA-499 in diagnosing AMI.

Our meta-analysis demonstrated a high degree of heterogeneity in the sensitivity (I^2^=98.13%, 95% CI [98.13, 98.78]) and specificity (I^2^=97.99%, 95% CI [97.52, 98.45]) of the studies, both exceeding 50%, indicating the need for a random-effects model. Nevertheless, miRNA-499 exhibited high SEN (0.88) and SPE (0.97) for diagnosing AMI. Further, the AUC was 0.98, indicating an excellent accuracy. The pooled PLR was 29.78, the pooled NLR was 0.13, and the pooled DOR was 236.10. The high SEN and SPE values of miRNA-499 in diagnosing AMI indicate its robustness as a diagnostic biomarker. Consistent with previous research, our study reaffirms the elevated expression levels of circulating miRNA-499 in AMI patients compared to healthy individuals. The mechanisms underlying this phenomenon are being unraveled [31]. Researchers have found that miRNA-499 expression is significantly increased in the myocardial infarcted area in rats, with subsequent studies suggesting that miRNA-499 might be released from damaged cardiomyocytes, thereby suppressing cardiomyocyte apoptosis by inhibiting the expression of pdcd4 and pacs2 [32].

This meta-analysis has several limitations. The significant heterogeneity among the included studies could be attributed to differences in sample sizes and sources, geographical regions, and publication years. As the number of studies included was restricted, a meta-regression analysis was not conducted to ascertain the origins of heterogeneity. Additionally, varying thresholds across studies may affect the reliability and accuracy of our meta-analysis. Furthermore, although no significant publication bias was detected, the likelihood of publication bias should not be completely disregarded as studies reporting positive results are often more likely to get published, potentially leading to an overestimation of the diagnostic accuracy. Despite these limitations, the high AUC value, combined with the substantial PLR and DOR, point towards the excellent diagnostic performance of miRNA-499, reinforcing its potential role as a reliable indicator for AMI.

This study suggests that circulating miRNA-499 is a valuable biomarker for diagnosing AMI. The incorporation of circulating miRNA-499 into clinical decision-making could potentially improve the accuracy of AMI treatment guidance. However, further research is required to establish standardized diagnostic criteria for its use, thus facilitating its integration into routine clinical practice. In light of these promising results, future research should focus on elucidating the exact mechanisms underlying the upregulation of miRNA-499 in AMI, and larger, more comprehensive studies are needed to validate our findings and overcome the limitations of the current study. Ultimately, the application of miRNA-499 as a diagnostic biomarker holds the potential to revolutionize AMI diagnosis and treatment, contributing significantly to improved patient outcomes.

## Conclusions

This meta-analysis substantiates the value of circulating miRNA-499 as a reliable biomarker for the diagnosis of acute myocardial infarction. The high pooled sensitivity, specificity, underscore the potential of miRNA-499 as a potential biomarker for AMI. The integration of this promising biomarker into routine clinical practice may enhance the accuracy of AMI diagnosis, thus improving patient prognosis and guiding effective therapeutic strategies. However, it’s essential to underscore the need for additional validation in larger and more diverse populations to ensure its reliability and accuracy before widespread adoption for optimizing its clinical utility and patient outcomes.

## Dodatak

### Conflict of interest statement

All the authors declare that they have no conflict of interest in this work.
